# Insights about circadian clock in glioma: From molecular pathways to therapeutic drugs

**DOI:** 10.1111/cns.13966

**Published:** 2022-09-06

**Authors:** Zongqi Wang, Gang Chen

**Affiliations:** ^1^ Department of Neurosurgery & Brain and Nerve Research Laboratory The First Affiliated Hospital of Soochow University Suzhou China; ^2^ Institute of Stroke Research Soochow University Suzhou China

**Keywords:** cell cycle, circadian clock, glioma, molecular pathway, therapeutic drug

## Abstract

Glioma is characterized as the most aggressive brain tumor that occurred in the central nervous system. The circadian rhythm is an essential cyclic change system generated by the endogenous circadian clock. Current studies found that the circadian clock affects glioma pathophysiology. It is still controversial whether the circadian rhythm disruption is a cause or an effect of tumorigenesis. This review discussed the association between cell cycle and circadian clock and provided a prominent molecular theoretical basis for tumor therapy. We illustrated the external factors affecting the circadian clock including thermodynamics, hypoxia, post‐translation, and microRNA, while the internal characteristics concerning the circadian clock in glioma involve stemness, metabolism, radiotherapy sensitivity, and chemotherapy sensitivity. We also summarized the molecular pathways and the therapeutic drugs involved in the glioma circadian rhythm. There are still many questions in this field waiting for further investigation. The results of glioma chronotherapy in sensitizing radiation therapy and chemotherapy have shown great therapeutic potential in improving clinical outcomes. These findings will help us further understand the characteristics of glioma pathophysiology.

## Graphical abstract

We summarized the connection between cell cycle clock and circadian clock. Previous studies reported that circadian clock proteins can affect cyclin‐CDK complex expression through p21, c‐Myc, and Wee1, and then participate in the regulation of the cell cycle. The external factors that influence the circadian clock and the internal characteristic changes affected by the circadian clock are listed aside.

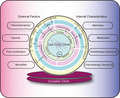

## 1 INTRODUCTION

Glioma is characterized as the most aggressive brain tumor type with a median survival period of only 14 months and a 5 years survival rate of less than 10% after diagnosis.^1^, ^2^, ^3^, ^4^ Current standard treatment strategies include complete or sub‐complete surgical resection, alkylating agent administration chemotherapy, and radiation therapy.^5^, ^6^ Alternative treatment can also be applied, including immunotherapy, targeted therapy, and tumor treatment fields (TT fields).^7^, ^8^, ^9^ Since glioma is characterized by rapid proliferation and aggressive infiltration, a considerable proportion of patients develop local recurrence and central nervous system metastasis.^10^

The circadian rhythm is an essential cyclic oscillation system generated by the endogenous circadian clock.^11^, ^12^ Essentially all mammalian organisms have developed circadian rhythms in order to synchronize social and physiological capabilities with explicit periodicity.^13^, ^14^ The control center of this circadian rhythm is located in the suprachiasmatic nucleus (SCN) of the hypothalamus, which regulates the rhythm production of various physiological functions.^15^, ^16^ Accumulating evidence reveals that the circadian rhythm has an impact on glioma pathophysiology. Temozolomide (TMZ) administration in the morning rather than evening exhibited longer overall survival (OS) with a significant first‐year restricted mean survival time difference.^17^ Low‐dose Bortezomib, the proteasome inhibitor in anticancer therapy, displayed higher efficacy when administered in tumor‐bearing animals at night compared to day/night administration.^18^ In mice injected with circadian clock gene *Bmal1* knocked‐down cells, a higher tumor growth rate was observed.^18^ Khan et al. revealed glioma‐genesis gene expression changes in the mouse brain after chronic alternating light–dark cycle exposure and suggested a potential connection between circadian clock disruption and glioma genesis risk.^19^ Wang F et al. revealed circadian clock disturbance promotes carcinogenesis through circadian clock gene *Period 2* (*Per2*) deregulation.^20^ Glioma also participates in circadian alteration and disruption. Athymic nude mice implanted with LN229 human glioma cells showed an increase in the endogenous period of the circadian clock and a slower resynchronization rate.^21^ SCN astrocytes modulate the circadian pacemaker via the regulation of glutamate levels.^22^, ^23^ Meanwhile, increased glutamate levels are characteristic of glioma, suggesting that dysregulation of proper glial function occurring in malignant tissue may impact timekeeping and clock synchronization.^24^, ^25^ In addition, molecules including TNF‐α and CCL2 involved in immune response affect the core circadian oscillator.^26^, ^27^, ^28^ Glioma alters the microenvironment to resist immune attack and cytokines and chemokines can be hijacked and then lead to circadian disruption.^26^, ^29^

Circadian activities associated with cerebrovascular reactivity and brain energy metabolism help to maintain central nerve system homeostasis.^30^, ^31^ Cerebral arteries possess a functional circadian clock and exhibit a diurnal rhythm in vasoreactivity to ATP.^30^ Brain metabolites are altered during sleep including acylcarnitines, hydroxylated fatty acids, phenolic compounds, and thiol‐containing metabolites.^31^ Disruption of these oscillations has been observed in cerebrovascular diseases.^32^, ^33^, ^34^ However, the circadian activities of glioma are still poorly understood, and circadian rhythm disruption is still controversial whether it is a cause or an effect of glioma genesis. Herein, we discussed the molecular connection between cell cycle and circadian rhythm clock, which are the major cyclic system, and focused on the external factors and internal characteristics associated with the glioma circadian clock. This review also summarized the molecular pathways and therapeutic drugs in glioma treatment by reviewing the circadian clock concerning differential molecular changes to explore the potential value in translational research.

## 2 CELL CYCLE AND CIRCADIAN CLOCK

Currently, the chemotherapeutic alkylating agent TMZ and radiation therapy regulate and inhibit cellular proliferation by destructing DNA replication.^35^, ^36^ DNA damage triggers the activation of cell cycle checkpoint pathways, which cause the cell cycle to be suspended, so the DNA repair machinery can detect and repair the damage.^37^ The DNA damage response (DDR) maintains genomic integrity while contributing to the resistance to chemotherapy and chemotherapy.^36^, ^37^, ^38^

The cell cycle checkpoint is an important cellular mechanism that prevents uncontrolled proliferation.^39^ The glioma cell cycle circulation has been well characterized, which consists of the rest/growth phase (G0/G1 phase), DNA synthesis phase (S phase), G2 phase, and mitosis phase (M phase).^40^ Arresting at G1/S or G2/M phase can effectively inhibit the cell proliferation process.^41^ The core cell‐cycle regulation mechanism depends on the cyclin‐dependent kinase (CDK) activation.^42^

The circadian clock oscillations rely on transcriptional‐translational auto‐regulatory feedback loops (TTFL), which are regulated by the activity of core molecular components.^43^ In mammals, the core clock genes govern circadian rhythm and consist of circadian locomotor output cycles kaput (*Clock*), brain and muscle ARN‐t like protein 1 (*Bmal1*), neuronal PAS domain protein 2 (*Npas2*), period protein family (*Per1, Per2*, and *Per3*), and cryptochrome family (*Cry 1* and *Cry 2*).^44^ The clock control genes include differentially expressed in chondrocyte family (*Dec1* and *Dec2*), nuclear receptor subfamily 1, group D member 1(*REV‐ERBα*), retinoic acid receptor‐related orphan receptor α (*RORα*), casein kinase 1 family (*CKIε* and *CKIδ*), and timeless (*Tim*).^44^ These circadian clock genes encode a highly conserved alkaline helix–loop–helix (bHLH) region that binds to DNA sequence “CANNTG” (E‐Box), which can be used as a core transcription enhancer.^45^ Conserved core components include the bHLH‐PAS transactivators, CLOCK/NPAS2, and BMAL1.^46^ PERs and CRYs inhibit CLOCK: BMAL1 heterodimer transcriptional activity and in turn their own expression, thus forming the negative feedback loop.^47^ An additional loop that is also induced by CLOCK: BMAL1 heterodimerization activates the rhythmic transcription of Rev‐erbα and Rorα.^48^ In addition, the CLOCK: BMAL1 heterodimer complex may be involved in activating a second alternative cycle consisting of REV‐ERBα and REV‐ERBβ which compete at the ROR‐binding elements.^49^ CKIε and CKIδ determine the circadian period length, through speed and rhythmicity regulation of PER1 and PER2 phosphorylation.^50^ DECs provide feedback and modulate CLOCK activity.^51^

**FIGURE 1:**
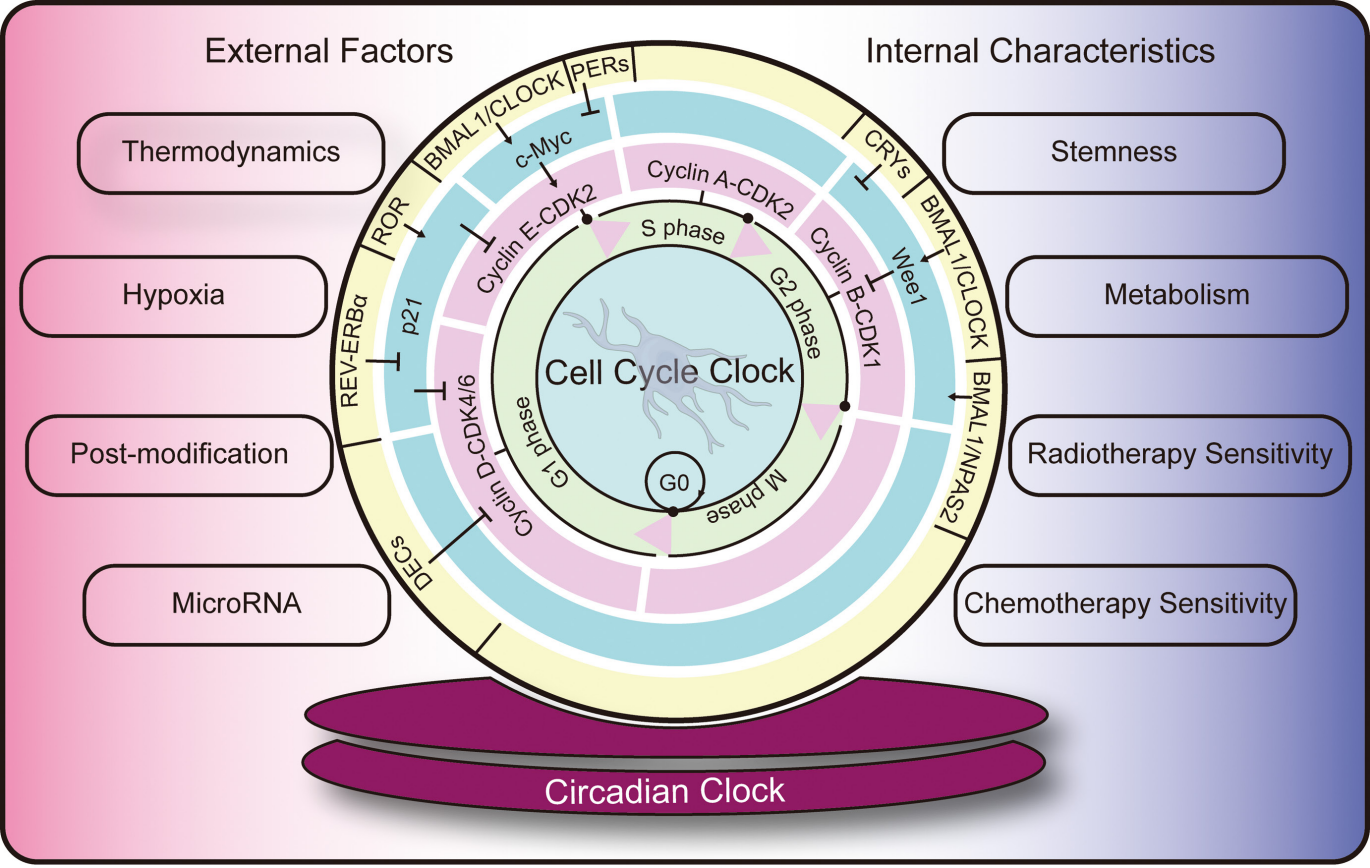
The connection between the cell cycle clock and the circadian clock. Previous studies reported that circadian clock proteins can affect the expression of the cyclin‐CDK complex through p21, c‐Myc, and Wee1, and then participate in the regulation of the cell cycle clock. The external factors that influence the circadian clock and the internal characteristic changes in glioma cells affected by the circadian clock are listed aside. BMAL1, Brain and muscle ARN‐t like protein 1; CLOCK, Circadian locomotor output cycles kaput; c‐Myc, Cellular‐myelocytomatosis viral oncogene; CRYs, Cryptochromes; DECs, Differentially expressed in chondrocytes; NPAS2, Neuronal PAS domain protein 2; p21, Cyclin‐dependent kinase inhibitor 1A; PERs, Period proteins; RORs, Retinoidrelated orphan nuclear receptors

Circadian rhythms persist in the presence or absence of environmental cycles because they are generated by endogenous mechanisms. Their periodic activity is composed of a periodically oscillating network formed by a series of rhythmically expressed proteins, and a variety of circadian clock factors affect the cell cycle by regulating the expression of the cyclin. In the G/S phase, REV‐ERBα inhibits p21 to promote cell progression,^52^ RORα activates p21 to inhibit cell progression,^53^ DEC1 inhibits cyclin D1,^54^ and CLOCK/BMAL1 negatively regulates c‐Myc^55^; in the G/M phase, BMAL1/CLOCK, BMAL1/NPAS2 or CRY1 acts on Wee1 to inhibit or activate glioma process^56^ (Figure 1). Therefore, elucidating the circadian rhythm effect on the cell cycle will provide a prominent molecular theoretical basis for tumor therapy.

## 3 EXTERNAL FACTORS AFFECTING GLIOMA CIRCADIAN CLOCK

### 3.1 Thermodynamics

Temperature compensation is characterized as the robust output of the circadian rhythm to the temperature fluctuation.^57^ Since the system level is temperature‐stable while individual components in the system are usually temperature‐sensitive under varying temperatures, the temperature compensation mechanism is peculiar.^58^ The *Cry1* gene expression fluctuated with the temperature while the other circadian genes showed no significant change.^58^ The temperature amplitude showed a tight connection between circadian and metabolic rhythms.

### 3.2 Hypoxia

Circadian dysregulation is exacerbated within the hypoxic tumor microenvironment (TME) in glioma.^59^, ^60^ The hypoxia‐inducible factor‐1α targets were negatively correlated with tumor suppressor *Clock* gene.^61^, ^62^ Loss of fidelity in timekeeping accelerates tumor development.^63^ Timekeeping fidelity loss or gain mechanism may offer a controllable switch for pharmaceutical research.^61^, ^62^, ^63^ Exploring interactions between the hypoxia and the circadian clock in TME may achieve a therapeutic advantage in hypoxia‐modifying compounds combined with first‐line treatments.

### 3.3 Post‐modification

Chromatin modification and post‐translational modification (PTM) of protein have a great impact on maintaining the periodic oscillation of the 24 h rhythm.^63^ Chromatin modification mainly regulates the transcriptional oscillation of rhythmic genes through histone acetylation, deacetylation, and methylation on the promoters of core clock genes.^64^ The *Clock* gene itself has acetyltransferase activity and can activate the transcription of the core clock gene through the CLOCK: BMAL1 heterodimer^65^; on the contrary, the inhibition of the core clock gene through the PERs/CRYs heterodimer is through regulating histone deacetylation and methylation.^66^ Phosphorylation, acetylation, and ubiquitination of protein PTM regulate activation of circadian oscillation.^65^, ^66^, ^67^ Tyrosine kinases CKIε and CKIδ regulate nucleus/cytoplasm transfer through phosphorylation of the core clock elements.^68^ CLOCK acetyltransferase activity can act on the lysine residues of BMAL1 to regulate the BMAL1 acetylation, which in turn influences the CLOCK: BMAL1 heterodimer combination.^69^ Protein ubiquitination regulates the core clock proteins' stability and thus affects circadian rhythms.^70^ IRE1a endoribonuclease could lead to PER1 degradation through cleaving the *Per1* mRNA.^71^ Differential expression of circadian genes in cancer or normal cells may thus provide a molecular theoretical basis for glioma chronotherapy.

### 3.4 MicroRNA

Small non‐coding RNAs, including MicroRNAs, participate in glioma pathophysiology processes including proliferation, invasion, survival, angiogenesis, and cancer metastasis.^72^ There have been reports that miR‐124 was downregulated in glioma.^73^ MiR‐124 decreasing may be responsible for *Clock* gene expression increasing, indicating its potential therapeutic values in glioma chronotherapy.^73^ miR‐7239‐3p secreted by M2 microglial exosomes is recruited in the TME.^74^ MiR‐7239‐3p in M2 microglial exosomes, not M1 type, inhibits *Bmal1* expression, promotes proliferation, and reduces apoptosis of glioma cells.^74^ MicroRNAs participate in circadian regulation and act as either suppressors or promoters.^72^

## 4 INTERNAL CHARACTERISTICS OF THE GLIOMA CIRCADIAN CLOCK

### 4.1 Stemness

Disruption of the circadian clock impairs glioma stem cells (GSCs) stemness in glioblastoma (GBM).^75^ *Bmal1* or *Clock* downregulation in GSCs induced cell cycle arrest and apoptosis.^76^ Epithelial‐mesenchymal transition (EMT) is a core event in promoting tumor cell metastasis, invasiveness, and GSCs populations. The specific phase activation in the *Per2* gene is a potential target for treatments that may suppress EMT, minimize GSCs, and limit tumor metastasis.^77^ *Per2* gene expression was enriched within C6 glioma tumor spheres but not in monolayer cell culture, suggesting that cell interactions or TME enable circadian timing.^51^

### 4.2 Metabolism

The molecular circadian clock circadian showed a tight connection with the metabolic/redox oscillator, which presents in organs, tissues, and individual cells.^46^ The molecular rhythm disruption may cause metabolic disorders.^46^ The synthesis and degradation of glycerophospholipids (GPLs) exhibit an oscillation in metabolisms with a 24 h periodicity.^78^ Moreover in quiescent cells, the *Per1* gene is tightly involved in GPL synthesis regulation.^79^ While in proliferating cells, targeting time‐dependent high‐level redox and low‐level GPL state revealed a potential efficient therapeutic pathway in chemotherapy.^80^ Constant light, which leads to circadian disruption, promotes anabolic metabolism.^81^

Under circadian disruption, more macrophages were recruited in the TME, genes involved in lipogenesis, and glucose uptake was upregulated.^79^, ^81^

### 4.3 Chemotherapy sensitivity

The chemotherapeutic alkylating agent TMZ and radiation therapy regulate and inhibit cellular proliferation by destructing DNA replication.^35^, ^36^ Chronotherapy affects the TMZ administration sensitivity of murine GBM tumor cells. Optimized TMZ administration time which is near the daily peak *Bmal1* expression maximized DDR, apoptosis activation, and growth inhibition.^82^ *Bmal1‐*deficient cells revealed circadian rhythm disruption in gene expression, TMZ‐induced apoptosis, and growth inhibition. Optimizing TMZ administration time in GBM treatment to daily rhythms should be evaluated in prospective clinical trials.^82^

### 4.4 Radiation therapy sensitivity

PERs participate in regulating the circadian rhythm in mammalian organisms. High *Per1* and *Per2* expression were associated with increased sensitivity to irradiation in glioma tissue.^83^ Following exposure to irradiation, higher *Per1* expression levels lead to serious DNA damage while the expression of important checkpoints in DNA damage, such as CHK2 and P53, increased.^84^ *Cry2* mRNA and protein levels exhibit 8 h periodicity in glioma tissue compared to 24 h in normal brain tissue. Higher *Cry2* expression in glioma tissues was in association with increased cell proliferation and irradiation resistance.^85^ Silencing *Clock* downregulated c‐Myc and Cyclin B1 and led to apoptosis and cell cycle arrest after irradiation.^86^

## 5 THE MOLECULAR PATHWAYS INVOLVED IN GLIOMA CIRCADIAN CLOCK

The key molecules of the circadian clock are involved in proliferation, invasion, migration, and tumorigenesis. The pathways involving circadian clock‐related molecules are summarized in Table 1.

**TABLE 1:** Research Characteristics of Glioma Circadian Clock Participating Pathways

Author	Publishing Time	Tissue/Cell Lines	Interference	Participating Pathways	Main Effects
Jarabo P et al.^88^	2022	Drosophila glioblastoma model	UAS‐*cry RNAi* fly stocks	PI3K pathway	PI3K pathway regulates CRY expression in glioblastoma cells, and in turn, CRY is necessary and sufficient to promote Myc accumulation in glioblastoma cells.
Gowda P et al.^106^	2021	A172, LN‐18	siRNA‐mediated gene silencing	IL‐1β‐mediated Inflammation	Lactate‐ IL‐1β autoregulatory circuit drives *Clock‐Bmal1* in glioma, and DHA and IL‐1β behave as *Clock‐Bmal1* targets; *Clock‐Bmal1* transcriptional network regulates genes associated with glioma progression
Gao Y et al.^93^	2021	U87‐MG	LVX‐IDH1‐mCMV‐ZsGreen‐PGK‐Puro and pLVX‐IDH1(MUT)‐mCMV‐ZsGreen‐PG K‐Puro lentiviruses	TGF‐β/Smad	*IDH1* R132H mutation may alter the cell cycle and biological rhythm genes in U87‐MG cells through the TGF‐β/Smad signaling pathway.
Ma D et al.^97^	2020	Human astrocyte, U87‐MG, U251, glioma stem cells. Intracranial implantation into male Balb/c male nude mice	Lentiviruses packaged in pGMLV‐Pe1‐Per2 for *Per2* overexpression	Wnt/β‐catenin	*Per2* overexpression could induce glioma stem cell arrest at the G0/G1 phase and suppress glioma proliferation, stemness and invasion ability in vitro and in vivo. Subsequently, the Wnt/β‐catenin signaling pathway was identified as the target of PER2 in glioma stem cells.
Gwon DH et al.^90^	2020	U87‐MG	Adenovirus‐mediated ectopic expression of Bmal1	cyclin B1‐mediated apoptosis	BMAL1 suppresses proliferation, migration, and invasion of U87‐MG cells by downregulating cyclin B1, phospho‐AKT, and MMP‐9. Downregulation of *cyclin B1* increased early and late apoptosis due to changes in the levels of BAX, BCL2, and cleaved Caspase‐3.
Goldsmith CS et al.^105^	2019	Human astrocyte; IM3	VX‐745 for p38 MAPK inhibition	p38 MAPK	Inhibition of p38 MAPK activity in IM3 cells at the time of day when the levels are normally low in human astrocytes under control of the circadian clock, significantly reduced IM3 invasiveness.
Yu M et al.^89^	2018	LN‐18; U118‐MG; primary human astrocytes	siRNA‐mediated gene silencing	PI3K/AKT axis; actin nucleation and polymerization	AXL mediated partially the regulatory effects of NR1D2 on PI3K/AKT axis and promoted proliferation, migration, and invasion of glioblastoma cells. Besides, *Rev‐erbβ* knockdown remarkably impaired the maturation of focal adhesion and assembly of F‐actin, along with downregulated phospho‐FAK, and proteins involved in actin nucleation and polymerization (phospho‐RAC1/CDC42, WAVE and PFN2).
Zhanfeng N et al.^103^	2016	U343; subcutaneous implantation into male Balb/c male nude mice	Per2 downregulation modified using a lentiviral transfection of shRNA	MDM2‐TP53	PER2 downregulation inhibits glioma cell apoptosis by activating the MDM2‐TP53 pathway
Jung CH et al^91^	2013	U251	siRNA‐mediated gene silencing	PI3K‐AKT‐MMP‐2	Bmal1 suppresses cancer cell invasion by blocking the PI3K‐AKT‐MMP‐2 signaling pathway.

Abbreviations: AXL, AXL receptor tyrosine kinase; BAX, Bcl2‐associated X protein; BCL2, B‐cell lymphoma 2; BMAL1, Brain and muscle ARN‐t like protein 1; Cdc42, Cell division cycle 42; Clock, Circadian locomotor output cycles kaput; Cry, Cryptochrome; DHA, Docosahexaenoic acid; FAK, Focal adhesion kinase; IL‐1β, Interleukin‐1 beta; MAPK, Mitogen‐activated protein kinases; Myc, Cellular‐myelocytomatosis viral oncogene; MDM2, Murine double minute 2; MMP‐2, metalloproteinase‐2; MMP‐9, metalloproteinase‐9; p21, Cyclin‐dependent kinase inhibitor 1A; PER2, Period 2; PFN2, Profilin 2; PI3K, Phosphatidylinositide 3 kinase; TGF‐β, Transforming growth factor‐beta; TP53, Tumor protein P53; WAVE, Wiskott Aldrich syndrome protein; Wnt, Wingless‐Type MMTV Integration Site Family.

### 5.1 PI3K/AKT pathway

Protein kinase B (PKB/Akt) activation via phosphatidylinositol 3‐kinase (PI3K) is highly related to tumorigenesis.^87^ In glioblastoma cells, the PI3K pathway regulates *Cry* expression. CRY is necessary and sufficient to promote the accumulation of oncogene Myc.^88^ PI3K/AKT pathway could also be regulated via tyrosine kinase AXL, which is the transcriptional target of REV‐ERBβ in glioblastoma cells.^89^ The core circadian clock gene *Bmal1* knockdown elevated cell invasion and migration through phosphorylated‐AKT and matrix metalloproteinase‐2 (MMP‐2) accumulation.^90^ BMAL1 is characterized as a tumor suppressor, which is capable of suppressing cancer cell growth and invasiveness.^91^ Recent studies demonstrated that there is a tight molecular connection between circadian rhythms and glioma genesis via PI3K/AKT pathway.

### 5.2 TGF‐β/Smad

The TGF‐β pathway participates in many cellular processes, including cell proliferation, invasion, migration, and extracellular matrix remodeling.^92^ TGF‐β is also an essential influencing factor of the physiological circadian clock rhythms. TGF‐β expression upregulation by adenovirus can induce *Bmal1* and *Npas2* expression significantly.^93^ TGF‐β binds and activates the serine–threonine kinase complex membrane receptor to phosphorylate Smad2 and Smad3; after phosphorylation, accumulation of Smad proteins in the nucleus forms complexes with transcription factors Smad4 to regulate transcription.^94^ *IDH1 R132H* mutation affected the TGF‐β/Smad signaling pathway. The circadian clock genes *Bmal1, Clock, Pers,* and *Crys* expression levels were significantly decreased. Those of the Smad signaling pathway genes *Smad2, Smad3,* and Smad*2‐3* were decreased, while phosphorylated *(p)‐Smad2, p‐Smad3,* and *Smad4* were increased.^93^

### 5.3 Wnt/β‐catenin

The WNT/beta‐catenin pathway induces the genes transcription involved in cell proliferation, cell invasiveness, nucleotide synthesis, tumor growth, and angiogenesis.^95^ WNT/β‐catenin signaling upregulation also induces molecular differential changes in core metabolic enzymes that modify their thermodynamics behavior.^96^ This leads to pyruvate dehydrogenase kinase 1 (PDK1) and monocarboxylate lactate transporter 1 (MCT1) activation.^96^ Consequently, phosphorylation of PDK1 inhibits the pyruvate dehydrogenase complex, which leads to aerobic glycolysis despite the oxygen availability, named the Warburg effect.^96^ In glioma cells, the Wnt/β‐catenin signaling pathway was identified as the target of PER2 in GSCs.^97^ Subsequently, downstream molecular PPARγ is downregulated, resulting in abnormalities in the regulation of circadian rhythms and destruction of circadian clock genes.^98^ These results indicated that PER2 plays a critical role in regulating the stemness of GSCs via the WNT/β‐catenin signaling pathway.

### 5.4 TP53

The MDM2/TP53 pathway is an important pathway for the occurrence and development of tumors. It is well characterized that TP53 is an important tumor suppressor gene.^99^ About 50% of tumors have TP53 mutations, resulting in the inactivation of its function.^100^ Murine double minute 2 (MDM2) is a key negative regulator of p53 and can mediate the degradation of TP53.^101^ MDM2 inhibitors can block TP53 degradation by inhibiting the function of MDM2, possibly restoring the function of TP53 protein.^102^ Lowering *Per2* expression reduces DNA damage response and cell death following low‐dose X‐ray irradiation. *Per2* was associated with increased activity of TP53 and participating in the DNA destruction damage during TP53‐mediated apoptosis.^103^

### 5.5 P38 MAPK

The p38 MAPK expression and activity increase are correlated with poor clinical prognosis, including GBM multiforme; however, the lethal toxicity of p38 MAPK inhibitors limits their therapeutic use.^104^ The phosphorylated p38 MAPK protein level was reduced in *Clock*‐deficient cells.^104^ The p38 MAPK activity inhibition with specific inhibitor VX‐745 led to cell‐type‐specific periodical changes in the molecular clock, indicating potential therapeutic use of VX‐745 in glioma chronotherapy.^104^ Under the control of the circadian clock, the p38 MAPK activity inhibition in invasive IM3 cells at the time point when the levels are normally low in human astrocytes significantly reduced IM3 invasiveness.^104^

### 5.6 Lactate‐IL‐1β‐Clock Loop

A desynchronized circadian rhythm in tumors is coincident with aberrant inflammation and dysregulated metabolism.^105^ The increase in tumor metabolite lactate results in the cytokine interleukin‐1β (IL‐1β) upregulation leading to inflammatory.^106^ IL‐1β was correlated with elevated levels of the core circadian regulators *Clock* and *Bmal1* ^106^. Lactate‐IL‐1β interaction positively affects the recruitment of CLOCK: BMAL1 to these E‐box sites in the nucleus.^107^ Lactate‐IL‐1β‐Clock Loop (LIC loop) was found to be correlated with the OS of patients.^107^

## 6 THE THERAPEUTIC DRUGS INVOLVED IN GLIOMA CIRCADIAN CLOCK

**FIGURE 2:**
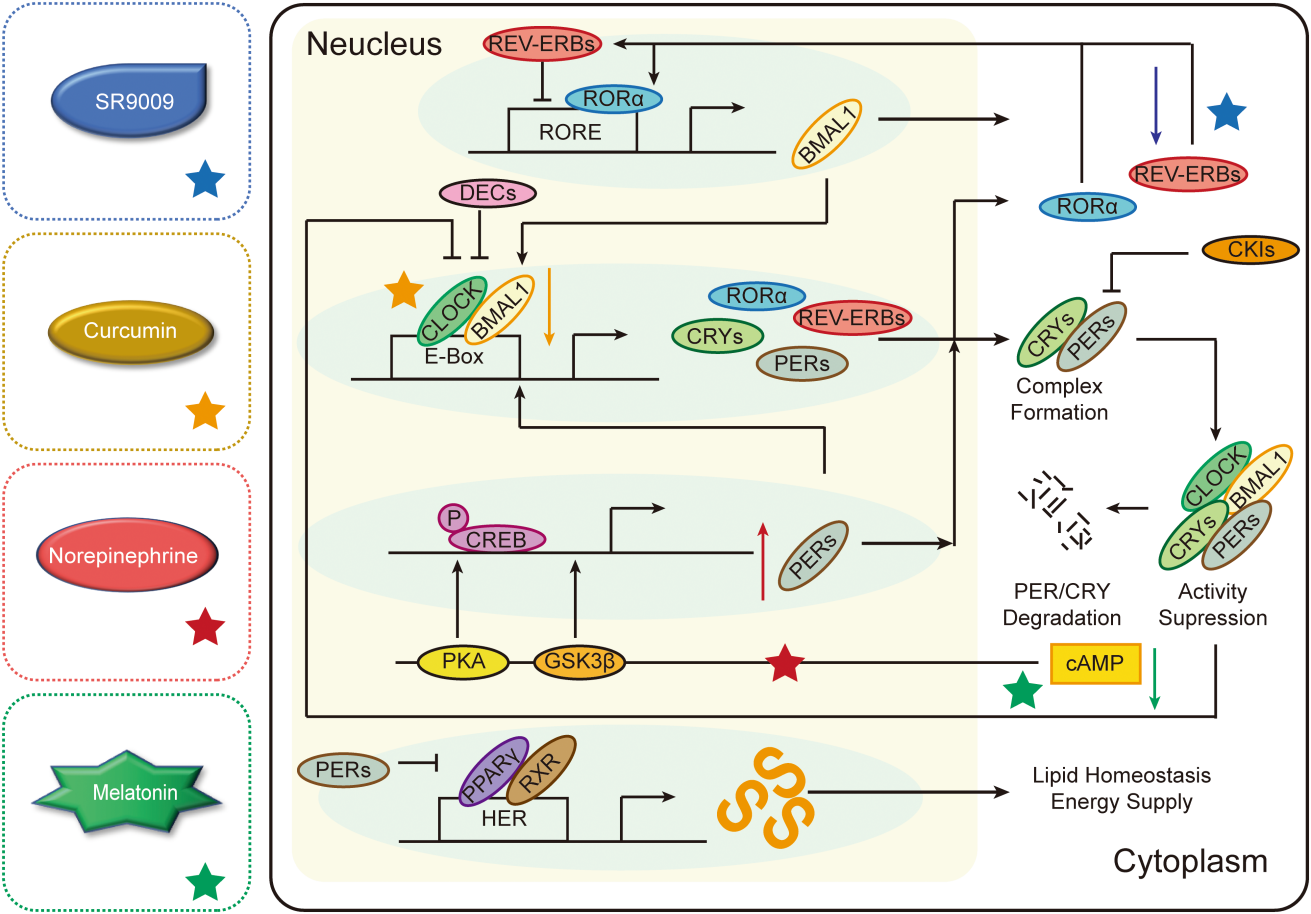
Molecular oscillation and therapeutic drugs of glioma. Norepinephrine, curcumin, AE lectin, and REV‐ERBs inhibitor SR9009 are involved in the circadian clock pathways. BMAL1, Brain and muscle ARN‐t like protein 1; CKIs, casein kinase 1 family; CLOCK, Circadian locomotor output cycles kaput; c‐AMP: Cellular‐myelocytomatosis viral oncogene; CREB, cAMP‐response element binding protein; CRYs, Cryptochromes; PERs, DECs, Differentially expressed in chondrocytes; Period proteins; PKA, protein kinase A; RORs, Retinoidrelated orphan nuclear receptors; GSK3β, Glycogen synthase kinase 3β; PPARγ, peroxisome proliferator‐activated receptor gamma

Current research methods for therapeutic drugs targeting the circadian clock mainly fall into two directions. One class of drugs that are widely used in clinical practice, including TMZ mentioned above, focuses on finding the optimal administration time for treatment. The cancer chronotherapy drug administration thus has been studied on in vivo animal, and potentially represents the combined effects both of time‐dependent pharmacokinetics and pharmacodynamics phenomena. Another class of therapeutic drugs regulates the proliferation of tumor cells by interfering with core proteins or substrates in the circadian clock (Figure 2). The two classes of drugs are reviewed in this section.

### 6.1 1A‐116

The 1A‐116 (ZINC69391) compound was recently exhibited as a novel drug in glioblastoma and other tumors treatment.^108^, ^109^ Both in LN229 and U‐87 glioma cell lines, 1A‐116 specifically inhibited Rac Family Small GTPase 1 (RAC1) activation to guanine exchange factors such as T‐lymphoma invasion and metastasis‐inducing protein 1 (Tiam1), by interacting with the Trp56 residue.^110^, ^111^ The effectiveness of 1A‐116 could be further optimized by finding the optimized time for the administration. The higher efficacy of 1A‐116 was observed at low BMAL1 expression in glioblastoma cells and a differential OS was found when applying 1A‐116 at Zeitgeber times 12 (ZT 12) to glioma‐bearing nude mice.^112^ The time‐dependent pharmacological application of 1A‐116 is a feasible strategy to improve OS.

### 6.2 Melatonin

Melatonin (N‐acetyl‐5‐methoxytryptamine, CAS#: 73–31‐4) is a naturally synthesized hormone involved in the biological clock, circadian rhythm, and reproductive physiology.^113^ It is produced by the pineal gland and acts on specific receptors and has an important role in overall energy metabolism.^114^ Melatonin is a powerful scavenger of reactive oxygen and nitrogen species (ROS/RNS), and also acts as a stimulator of the antioxidant enzymes leading to a DNA damage decrease.^115^ However, high melatonin (mM range) concentrations have been shown to impair the invasion and migration ability of human glioma cell lines and reduce the viability, while lower concentrations did not produce significant results.^115^, ^116^ Human MT1 and MT2 melatonin receptors share 55% amino acid sequence similarity, but the two receptors play opposite roles in glioma progression.^117^ MT1 activation and MT2 depression exhibit robust antitumor effects and interfere with the proliferation and metabolism of glioma stem cells.^118^ Melatonin increases calmodulin degradation by direct binding while redistributing calmodulin, thereby arresting the S/M phases and reentry of G0 quiescent cells into the cell cycle.^119^ Melatonin acts antitumor effect via inhibiting the production of cAMP production, resulting in diminished linoleic acid uptake and 15‐lipoxygenase oxidation serving as an energy source for tumor growth.^120^

### 6.3 Curcumin

Curcumin (diferuloylmethane, CAS#:458–37‐7) is a promising phytochemical that can be administered in glioma therapy.^121^ Administration of curcumin can alter molecular circadian timing within cells. The prominent target, BMAL1, is the core gene in molecular oscillators that generates circadian rhythms.^122^ Studies reported that curcumin can affect *STAT, PPAR‐γ*, and *NF‐kB* expression within two interacted molecular timing loops.^121^, ^122^ BMAL1 is activated by curcumin via PPAR‐γ stimulation.^108^ Research has also shown that curcumin activates sirtuin 1, and binds to CLOCK: BMAL1 heterodimer to promote the deacetylation and degradation of PER2.^69^ Although no effect on the circadian mechanism has been reported, 10 μM curcumin treatment did disrupt a single circadian oscillator within the clock unit or coupling between circadian clocks in apoptosis.^123^ During anticancer treatment, curcumin or its analogs should be administered to tumor cells at the optimal stage in maximizing efficacy after determining the circadian phase.

### 6.4 Norepinephrine

Norepinephrine (NA, CAS#: 51–41‐2) is primarily located in the brain stem and is involved in behaviors including sleep and awakening.^124^ NA can act on the biological pineal region cells pinealocytes. Promoting cAMP activates arylalkylamine N‐acetyltransferase (AANAT), which is the melatonin synthesis rate‐limiting enzyme.^124^ NA administration led to increasing in *Per1* mRNA expression via β2‐adrenergic receptors.^125^, ^126^ Furthermore, this same reaction might be involved in the activities of both protein kinase A (PKA) and the protein tyrosine kinase Src family.^127^ The PKA‐CREB signaling cascade coupled with β2‐adrenoceptors has been shown to play an essential role in the regulation of clock genes including *Per1* in cerebellar granule cells and chondrocytes.^124^

### 6.5 REV‐ERB agonist

REV‐ERBs are essential components participating in the circadian clock. The novel drugs SR9009 and SR9011, agonists of REV‐ERBs, are specifically lethal to cancer cells and oncogene‐induced senescent cells and do not affect the viability of normal cells or tissues.^128^ SR9009 treatment decreased ROS levels and increased the level of lipid droplets, whereas the combined treatment with Bortezomib showed additive or synergistic effects between both drugs in T98G cells.^128^ The autophagy regulation and de novo lipogenesis by REV‐ERB agonist administration have a great impact on evoking an apoptotic response in malignant cells.^125^ Notably, these REV‐ERB agonists showed selective anticancer properties, impaired glioblastoma growth in vivo*,* and improved survival without causing overt toxicity in mice.^125^ SR9009 and SR9011 anticancer activity affects several oncogenic drivers, including HRAS, BRAF, and PIK3CA, and persists in the absence of TP53 and under hypoxic conditions which need further illustration.

## 7 FUTURE PERSPECTIVE

This review discussed the molecular connection between cell cycle and circadian clock, illustrated the external factors and internal characteristics concerning the circadian clock in glioma, and summarized the molecular pathways and the therapeutic drugs involved in the glioma circadian rhythm. However, the results of chronotherapy for temozolomide and radiation therapy in glioma are currently controversial. Damato AR et al. reported no significant difference in adverse events, quality of life, or OS in prospective randomized TMZ chronotherapy trials.^123^ For high‐grade glioma patients, radiotherapy treatment time of the day (RT‐TTD) did not influence progression‐free survival and OS between patients treated in the morning or afternoon.^129^ The heterogeneity of each study is worth our attention. Besides tumor grade as a confounding factor should be analyzed respectively in the subsequent study. There is still a lack of multi‐center, double‐blinded random control trials to further elaborate on the influence of the circadian clock in glioma patients.

There are still many questions in this field waiting for further investigation. From the perspective of etiology, whether the changes in circadian rhythm are an essential physiological factor in glioma genesis; From the perspective of treatment, how to accurately identify the patient's circadian clock during clinical treatment, and whether the treatment regimen is customized based on the circadian clock of the patient; The therapeutic and adverse effects of changing the circadian clock of glioma patients must be considered seriously. Currently, experimental research performed serum shock procedures to synchronize cells in vitro^107^. In vivo, animals were kept at an inverse 12 h light/12 h dark cycle, facilitating adaptation to the environmental cycle of day and night.^21^, ^112^ Besides, the different TME between in vivo and in vitro are worthy of attention. The circadian clock of laboratory animals may differ significantly from those of humans. Since mice are nocturnal animals, and their active and rest phases are out of phase with humans, there are obvious differences in circadian rhythms in mice and humans.^130^ In addition, tumor cell‐intrinsic circadian rhythms can regulate TMZ cytotoxicity in mice, which showed a strong correlation between drug sensitivity and circadian rhythm,^82^ while the effect of TMZ in glioma chronotherapy treatment is still controversial and needs further large‐population‐based trials for validation.^17^, ^123^ Despite being more complex and expensive than murine models, nonhuman primates share more closely activity patterns with humans, which may provide stronger evidence support for chronotherapy research.^131^ The generalization of these conclusions to other species will require additional systematic study. Besides, several non‐invasive imaging modalities, including quantitative parametric images of O‐(2‐[F]fluoroethyl)‐L‐tyrosine kinetics analysis and MRI approach for noninvasive TME visualization, have been developed for quantitatively assessing the oxygen and energy metabolism within glioma or the microenvironment surrounding glioma.^132^, ^133^ Future studies should include the longitudinal measurement of glioma metabolism, determining how circadian activities may alter the metabolic profile of glioma. Regardless, the results of glioma chronotherapy in sensitizing radiation therapy and chemotherapy are exciting. These findings will help us further understand this pathological process in chronotherapy.

## FUNDING INFORMATION

This work was supported by the National Natural Science Foundation of China under Grant [82002643]; China Postdoctoral Science Foundation under Grant [2019 M651954].

## CONFLICT OF INTEREST

The authors declare that there are no conflicts of interest.

## Data Availability

The data that support the findings of this study are available from the corresponding author upon reasonable request.

## References

[1] Liu Y , Shi Y , Wu M , et al. Hypoxia‐induced polypoid giant cancer cells in glioma promote the transformation of tumor‐associated macrophages to a tumor‐supportive phenotype. CNS Neurosci Ther. 2022;28:1326‐1338. 35762580 10.1111/cns.13892 PMC9344088

[2] Liu T , Ma W , Xu H , et al. PDGF‐mediated mesenchymal transformation renders endothelial resistance to anti‐VEGF treatment in glioblastoma. Nat Commun. 2018;9(1):3439. 30150753 10.1038/s41467-018-05982-z PMC6110798

[3] Huang H , Yang G , Zhang W , et al. A deep multi‐task learning framework for brain tumor segmentation. Front Oncol. 2021;11:690244. 34150660 10.3389/fonc.2021.690244 PMC8212784

[4] Grande S , Palma A , Ricci‐Vitiani L , et al. Metabolic heterogeneity evidenced by mrs among patient‐derived glioblastoma multiforme stem‐like cells accounts for cell clustering and different responses to drugs. Stem Cells Int. 2018;2018:3292704‐3292716. 29531533 10.1155/2018/3292704 PMC5835274

[5] Schloss J , Lacey J , Sinclair J , et al. A phase 2 randomised clinical trial assessing the tolerability of two different ratios of medicinal cannabis in patients with high grade gliomas. Front Oncol. 2021;11:649555. 34094937 10.3389/fonc.2021.649555 PMC8176855

[6] Pei Y , Liu KW , Wang J , et al. HDAC and PI3K antagonists cooperate to inhibit growth of MYC‐driven medulloblastoma. Cancer Cell. 2016;29(3):311‐323. 26977882 10.1016/j.ccell.2016.02.011 PMC4794752

[7] Wu H , Yang L , Liu H , et al. Exploring the efficacy of tumor electric field therapy against glioblastoma: an in vivo and in vitro study. CNS Neurosci Ther. 2021;27(12):1587‐1604. 34710276 10.1111/cns.13750 PMC8611775

[8] Tan YQ , Li YT , Yan TF , et al. Six immune associated genes construct prognostic model evaluate low‐grade glioma. Front Immunol. 2020;11:606164. 33408717 10.3389/fimmu.2020.606164 PMC7779629

[9] Cai H , Yu Y , Ni X , et al. LncRNA LINC00998 inhibits the malignant glioma phenotype via the CBX3‐mediated c‐Met/Akt/mTOR axis. Cell Death Dis. 2020;11(12):1032. 33268783 10.1038/s41419-020-03247-6 PMC7710718

[10] Nelson SL , Proctor DT , Ghasemloonia A , et al. Vibrational profiling of brain tumors and cells. Theranostics. 2017;7(9):2417‐2430. 28744324 10.7150/thno.19172 PMC5525746

[11] Lu M , Huang L , Tang Y , et al. ARNTL2 knockdown suppressed the invasion and migration of colon carcinoma: decreased SMOC2‐EMT expression through inactivation of PI3K/AKT pathway. Am J Transl Res. 2020;12(4):1293‐1308. 32355542 PMC7191172

[12] Li X , Zhao H . Automated feature extraction from population wearable device data identified novel loci associated with sleep and circadian rhythms. PLoS Genet. 2020;16(10):e1009089. 33075057 10.1371/journal.pgen.1009089 PMC7595622

[13] Morley‐Fletcher S , Mairesse J , Van Camp G , et al. Perinatal stress programs sex differences in the behavioral and molecular chronobiological profile of rats maintained under a 12‐h light‐dark cycle. Front Mol Neurosci. 2019;12:89. 31118884 10.3389/fnmol.2019.00089 PMC6504690

[14] Kuo J , Yuan R , Sanchez C , Paulsson J , Silver PA . Toward a translationally independent RNA‐based synthetic oscillator using deactivated CRISPR‐Cas. Nucleic Acids Res. 2020;48(14):8165‐8177. 32609820 10.1093/nar/gkaa557 PMC7430638

[15] Cohen DA , Wang W , Wyatt JK , Kronauer RE , Dijk DJ , Czeisler CA , Klerman EB Uncovering residual effects of chronic sleep loss on human performance. Sci Transl Med 2010;2(14):14ra3, 14ra13, 2. 10.1126/scitranslmed.3000458 PMC2892834 20371466

[16] Chuffa LGA , Lupi LA , Cucielo MS , Silveira HS , Reiter RJ , Seiva FRF . Melatonin promotes uterine and placental health: potential molecular mechanisms. Int J Mol Sci. 2019;21(1):300. 10.3390/ijms21010300 PMC6982088 31906255

[17] Damato AR , Luo J , Katumba RGN , et al. Temozolomide chronotherapy in patients with glioblastoma: a retrospective single‐institute study. Neurooncol Adv. 2021;3(1):vdab041. 33959716 10.1093/noajnl/vdab041 PMC8086242

[18] Wagner PM , Prucca CG , Velazquez FN , Sosa Alderete LG , Caputto BL , Guido ME . Temporal regulation of tumor growth in nocturnal mammals: In vivo studies and chemotherapeutical potential. FASEB J. 2021;35(2):e21231. 33428275 10.1096/fj.202001753R

[19] Khan S , Liu Y , Siddique R , Nabi G , Xue M , Hou H . Impact of chronically alternating light‐dark cycles on circadian clock mediated expression of cancer (glioma)‐related genes in the brain. Int J Biol Sci. 2019;15(9):1816‐1834. 31523185 10.7150/ijbs.35520 PMC6743288

[20] Wang F , Luo Y , Li C , Chen L . Correlation between deregulated expression of PER2 gene and degree of glioma malignancy. Tumori. 2014;100(6):e266‐e272. 25688509 10.1700/1778.19292

[21] Duhart JM , Brocardo L , Caldart CS , Marpegan L , Golombek DA . Circadian alterations in a murine model of hypothalamic glioma. Front Physiol. 2017;8:864. 29163208 10.3389/fphys.2017.00864 PMC5670357

[22] Leone MJ , Beaule C , Marpegan L , Simon T , Herzog ED , Golombek DA . Glial and light‐dependent glutamate metabolism in the suprachiasmatic nuclei. Chronobiol Int. 2015;32(4):573‐578. 25798929 10.3109/07420528.2015.1006328

[23] Brancaccio M , Patton AP , Chesham JE , Maywood ES , Hastings MH . Astrocytes control circadian timekeeping in the suprachiasmatic nucleus via glutamatergic signaling. Neuron. 2017;93(6):1420‐1435. 28285822 10.1016/j.neuron.2017.02.030 PMC5376383

[24] de Groot J , Sontheimer H . Glutamate and the biology of gliomas. Glia. 2011;59(8):1181‐1189. 21192095 10.1002/glia.21113 PMC3107875

[25] Robert SM , Sontheimer H . Glutamate transporters in the biology of malignant gliomas. Cell Mol Life Sci. 2014;71(10):1839‐1854. 24281762 10.1007/s00018-013-1521-z PMC3999209

[26] Christofides A , Kosmopoulos M , Piperi C . Pathophysiological mechanisms regulated by cytokines in gliomas. Cytokine. 2015;71(2):377‐384. 25458967 10.1016/j.cyto.2014.09.008

[27] Duhart JM , Brocardo L , Mul Fedele ML , Guglielmotti A , Golombek DA . CCL2 mediates the circadian response to low dose endotoxin. Neuropharmacology. 2016;108:373‐381. 27178133 10.1016/j.neuropharm.2016.05.005

[28] Leone MJ , Marpegan L , Duhart JM , Golombek DA . Role of proinflammatory cytokines on lipopolysaccharide‐induced phase shifts in locomotor activity circadian rhythm. Chronobiol Int. 2012;29(6):715‐723. 22734572 10.3109/07420528.2012.682681

[29] Cornelison RC , Yuan JX , Tate KM , et al. A patient‐designed tissue‐engineered model of the infiltrative glioblastoma microenvironment. NPJ Precis Oncol. 2022;6(1):54. 35906273 10.1038/s41698-022-00290-8 PMC9338058

[30] Durgan DJ , Crossland RF , Bryan RM Jr . The rat cerebral vasculature exhibits time‐of‐day‐dependent oscillations in circadian clock genes and vascular function that are attenuated following obstructive sleep apnea. J Cereb Blood Flow Metab. 2017;37(8):2806‐2819. 27798273 10.1177/0271678X16675879 PMC5536790

[31] Vallianatou T , Lin W , Bechet NB , et al. Differential regulation of oxidative stress, microbiota‐derived, and energy metabolites in the mouse brain during sleep. J Cereb Blood Flow Metab. 2021;41(12):3324‐3338. 34293940 10.1177/0271678X211033358 PMC8669215

[32] Boltze J , Didwischus N , Merrow M , Dallmann R , Plesnila N . Circadian effects on stroke outcome ‐ did we not wake up in time for neuroprotection? J Cereb Blood Flow Metab. 2021;41(3):684‐686. 33337257 10.1177/0271678X20978711 PMC7907996

[33] Lo EH , Albers GW , Dichgans M , et al. Circadian biology and stroke. Stroke. 2021;52(6):2180‐2190. 33940951 10.1161/STROKEAHA.120.031742

[34] Wu L , Chan ST , Edmiston WJ 3rd , et al. Persistent CO2 reactivity deficits are associated with neurological dysfunction up to one year after repetitive mild closed head injury in adolescent mice. J Cereb Blood Flow Metab. 2021;41(12):3260‐3272. 34229511 10.1177/0271678X211021771 PMC8669283

[35] Ohba S , Yamashiro K , Hirose Y . Inhibition of DNA repair in combination with temozolomide or dianhydrogalactiol overcomes temozolomide‐resistant glioma cells. Cancers (Basel). 2021;13(11):2570. 34073837 10.3390/cancers13112570 PMC8197190

[36] McCord M , Steffens A , Javier R , Kam KL , McCortney K , Horbinski C . The efficacy of DNA mismatch repair enzyme immunohistochemistry as a screening test for hypermutated gliomas. Acta Neuropathol Commun. 2020;8(1):15. 32051040 10.1186/s40478-020-0892-2 PMC7017562

[37] Yang Q , Zhou Y , Chen J , Huang N , Wang Z , Cheng Y . Gene therapy for drug‐resistant glioblastoma via lipid‐polymer hybrid nanoparticles combined with focused ultrasound. Int J Nanomedicine. 2021;16:185‐199. 33447034 10.2147/IJN.S286221 PMC7802796

[38] Farhy C , Hariharan S , Ylanko J , et al. Improving drug discovery using image‐based multiparametric analysis of the epigenetic landscape. Elife. 2019;8:e49683. 31637999 10.7554/eLife.49683 PMC6908434

[39] Wanigasooriya K , Tyler R , Barros‐Silva JD , Sinha Y , Ismail T , Beggs AD . Radiosensitising cancer using phosphatidylinositol‐3‐kinase (PI3K), protein kinase B (AKT) or mammalian target of rapamycin (mTOR) inhibitors. Cancers (Basel). 2020;12(5):1278. 10.3390/cancers12051278 PMC7281073 32443649

[40] Chaudhury I , Koepp DM . Recovery from the DNA replication checkpoint. Genes (Basel). 2016;7(11):94. 10.3390/genes7110094 PMC5126780 27801838

[41] Wu J , Jiang H , Luo S , et al. Caspase‐mediated cleavage of C53/LZAP protein causes abnormal microtubule bundling and rupture of the nuclear envelope. Cell Res. 2013;23(5):691‐704. 23478299 10.1038/cr.2013.36 PMC3641598

[42] Izumi Y , Fujii K , Wien F , et al. Structure change from beta‐strand and turn to alpha‐helix in histone H2A‐H2B induced by DNA damage response. Biophys J. 2016;111(1):69‐78. 27410735 10.1016/j.bpj.2016.06.002 PMC4945303

[43] Chatterjee S , Angelakos CC , Bahl E , et al. The CBP KIX domain regulates long‐term memory and circadian activity. BMC Biol. 2020;18(1):155. 33121486 10.1186/s12915-020-00886-1 PMC7597000

[44] Ouyang D , Furuike Y , Mukaiyama A , Ito‐Miwa K , Kondo T , Akiyama S . Development and optimization of expression, purification, and atpase assay of kaic for medium‐throughput screening of circadian clock mutants in cyanobacteria. Int J Mol Sci. 2019;20(11):2789. 10.3390/ijms20112789 PMC6600144 31181593

[45] Lu C , Thompson CB . Metabolic regulation of epigenetics. Cell Metab. 2012;16(1):9‐17. 22768835 10.1016/j.cmet.2012.06.001 PMC3392647

[46] Satou R , Sato M , Kimura M , et al. Temporal Expression Patterns of Clock Genes and Aquaporin 5/Anoctamin 1 in Rat Submandibular Gland Cells. Front Physiol. 2017;8:320. 28588500 10.3389/fphys.2017.00320 PMC5440558

[47] Yoshitane H , Ozaki H , Terajima H , et al. CLOCK‐controlled polyphonic regulation of circadian rhythms through canonical and noncanonical E‐boxes. Mol Cell Biol. 2014;34(10):1776‐1787. 24591654 10.1128/MCB.01465-13 PMC4019033

[48] Lin Y , Wang S , Gao L , et al. Oscillating lncRNA Platr4 regulates NLRP3 inflammasome to ameliorate nonalcoholic steatohepatitis in mice. Theranostics. 2021;11(1):426‐444. 33391484 10.7150/thno.50281 PMC7681083

[49] Pudasaini A , Shim JS , Song YH , et al. Kinetics of the LOV domain of ZEITLUPE determine its circadian function in Arabidopsis. Elife. 2017;6:e21646. 28244872 10.7554/eLife.21646 PMC5370183

[50] Lee Y , Jang AR , Francey LJ , Sehgal A , Hogenesch JB . KPNB1 mediates PER/CRY nuclear translocation and circadian clock function. Elife. 2015;4:e08647. 10.7554/eLife.08647 PMC4597257 26319354

[51] Sharma VP , Anderson NT , Geusz ME . Circadian properties of cancer stem cells in glioma cell cultures and tumorspheres. Cancer Lett. 2014;345(1):65‐74. 24333739 10.1016/j.canlet.2013.11.009

[52] Sanchez DI , Gonzalez‐Fernandez B , Crespo I , et al. Melatonin modulates dysregulated circadian clocks in mice with diethylnitrosamine‐induced hepatocellular carcinoma. J Pineal Res. 2018;65(3):e12506. 29770483 10.1111/jpi.12506

[53] Roa SLR , Martinez EZ , Martins CS , Antonini SR , de Castro M , Moreira AC . Postnatal ontogeny of the circadian expression of the adrenal clock genes and corticosterone rhythm in male rats. Endocrinology. 2017;158(5):1339‐1346. 28324022 10.1210/en.2016-1782

[54] Liu Y , Wang L , Lin XY , et al. The transcription factor DEC1 (BHLHE40/STRA13/SHARP‐2) is negatively associated with TNM stage in non‐small‐cell lung cancer and inhibits the proliferation through cyclin D1 in A549 and BE1 cells. Tumour Biol. 2013;34(3):1641‐1650. 23423709 10.1007/s13277-013-0697-z

[55] Xu H , Gustafson CL , Sammons PJ , et al. Cryptochrome 1 regulates the circadian clock through dynamic interactions with the BMAL1 C terminus. Nat Struct Mol Biol. 2015;22(6):476‐484. 25961797 10.1038/nsmb.3018 PMC4456216

[56] Obi‐Ioka Y , Ushijima K , Kusama M , Ishikawa‐Kobayashi E , Fujimura A . Involvement of Wee1 in the circadian rhythm‐dependent intestinal damage induced by docetaxel. J Pharmacol Exp Ther. 2013;347(1):242‐248. 23892568 10.1124/jpet.113.203299

[57] Goda T , Hamada FN . Drosophila temperature preference rhythms: an innovative model to understand body temperature rhythms. Int J Mol Sci. 2019;20(8):1988. 10.3390/ijms20081988 PMC6514862 31018551

[58] Kurosawa G , Fujioka A , Koinuma S , Mochizuki A , Shigeyoshi Y . Temperature‐amplitude coupling for stable biological rhythms at different temperatures. PLoS Comput Biol. 2017;13(6):e1005501. 28594845 10.1371/journal.pcbi.1005501 PMC5464531

[59] Luan X , Guan YY , Liu HJ , et al. A tumor vascular‐targeted interlocking trimodal nanosystem that induces and exploits hypoxia. Adv Sci (Weinh). 2018;5(8):1800034. 30128230 10.1002/advs.201800034 PMC6097144

[60] Chang WH , Lai AG . Timing gone awry: distinct tumour suppressive and oncogenic roles of the circadian clock and crosstalk with hypoxia signalling in diverse malignancies. J Transl Med. 2019;17(1):132. 31014368 10.1186/s12967-019-1880-9 PMC6480786

[61] Texada MJ , Jorgensen AF , Christensen CF , et al. A fat‐tissue sensor couples growth to oxygen availability by remotely controlling insulin secretion. Nat Commun. 2019;10(1):1955. 31028268 10.1038/s41467-019-09943-y PMC6486587

[62] Lee J , Heo J , Kang H . miR‐92b‐3p‐TSC1 axis is critical for mTOR signaling‐mediated vascular smooth muscle cell proliferation induced by hypoxia. Cell Death Differ. 2019;26(9):1782‐1795. 30518907 10.1038/s41418-018-0243-z PMC6748132

[63] Xie N , Zhang L , Gao W , et al. NAD(+) metabolism: pathophysiologic mechanisms and therapeutic potential. Signal Transduct Target Ther. 2020;5(1):227. 33028824 10.1038/s41392-020-00311-7 PMC7539288

[64] Bellet MM , Sassone‐Corsi P . Mammalian circadian clock and metabolism ‐ the epigenetic link. J Cell Sci. 2010;123(Pt 22):3837‐3848. 21048160 10.1242/jcs.051649 PMC2972271

[65] Hosoda H , Kato K , Asano H , et al. CBP/p300 is a cell type‐specific modulator of CLOCK/BMAL1‐mediated transcription. Mol Brain. 2009;2:34. 19922678 10.1186/1756-6606-2-34 PMC2785803

[66] Sun C , Liu S , He C , et al. Crosstalk between the circadian clock and histone methylation. Int J Mol Sci. 2022;23(12):281‐298. 10.3390/ijms23126465 PMC9224359 35742907

[67] Haspel JA , Anafi R , Brown MK , et al. Perfect timing: circadian rhythms, sleep, and immunity ‐ an NIH workshop summary. JCI Insight. 2020;5(1):e131487. 10.1172/jci.insight.131487 PMC7030790 31941836

[68] Ogata H , Horie M , Kayaba M , et al. Skipping breakfast for 6 days delayed the circadian rhythm of the body temperature without altering clock gene expression in human leukocytes. Nutrients. 2020;12(9):2797. 10.3390/nu12092797 PMC7551061 32932677

[69] Zheng X , Zhao X , Zhang Y , et al. RAE1 promotes BMAL1 shuttling and regulates degradation and activity of CLOCK: BMAL1 heterodimer. Cell Death Dis. 2019;10(2):62. 30683868 10.1038/s41419-019-1346-2 PMC6347605

[70] Fan W , Chen X , Li C , et al. The analysis of deregulated expression and methylation of the PER2 genes in gliomas. J Cancer Res Ther. 2014;10(3):636‐640. 25313752 10.4103/0973-1482.138202

[71] Pluquet O , Dejeans N , Bouchecareilh M , et al. Posttranscriptional regulation of PER1 underlies the oncogenic function of IREalpha. Cancer Res. 2013;73(15):4732‐4743. 23752693 10.1158/0008-5472.CAN-12-3989 PMC3915716

[72] Ando H , Hirose M , Kurosawa G , Impey S , Mikoshiba K . Time‐lapse imaging of microRNA activity reveals the kinetics of microRNA activation in single living cells. Sci Rep. 2017;7(1):12642. 28974737 10.1038/s41598-017-12879-2 PMC5626736

[73] Li A , Lin X , Tan X , et al. Circadian gene clock contributes to cell proliferation and migration of glioma and is directly regulated by tumor‐suppressive miR‐124. FEBS Lett. 2013;587(15):2455‐2460. 23792158 10.1016/j.febslet.2013.06.018

[74] Li X , Guan J , Jiang Z , et al. Microglial exosome miR‐7239‐3p promotes glioma progression by regulating circadian genes. Neurosci Bull. 2021;37(4):497‐510. 33528793 10.1007/s12264-020-00626-z PMC8055789

[75] Huang W , Zhong Z , Luo C , et al. The miR‐26a/AP‐2alpha/Nanog signaling axis mediates stem cell self‐renewal and temozolomide resistance in glioma. Theranostics. 2019;9(19):5497‐5516. 31534499 10.7150/thno.33800 PMC6735392

[76] Dong Z , Zhang G , Qu M , et al. Targeting glioblastoma stem cells through disruption of the circadian clock. Cancer Discov. 2019;9(11):1556‐1573. 31455674 10.1158/2159-8290.CD-19-0215 PMC6983300

[77] De A , Beligala DH , Sharma VP , Burgos CA , Lee AM , Geusz ME . Cancer stem cell generation during epithelial‐mesenchymal transition is temporally gated by intrinsic circadian clocks. Clin Exp Metastasis. 2020;37(5):617‐635. 32816185 10.1007/s10585-020-10051-1

[78] Blazevits O , Bolshette N , Vecchio D , et al. MYC‐associated factor MAX is a regulator of the circadian clock. Int J Mol Sci. 2020;21(7):2294. 10.3390/ijms21072294 PMC7177918 32225100

[79] Guido ME , Monjes NM , Wagner PM , Salvador GA . Circadian regulation and clock‐controlled mechanisms of glycerophospholipid metabolism from neuronal cells and tissues to fibroblasts. Mol Neurobiol. 2022;59(1):326‐353. 34697790 10.1007/s12035-021-02595-4

[80] Wagner PM , Sosa Alderete LG , Gorne LD , et al. Proliferative glioblastoma cancer cells exhibit persisting temporal control of metabolism and display differential temporal drug susceptibility in chemotherapy. Mol Neurobiol. 2019;56(2):1276‐1292. 29881948 10.1007/s12035-018-1152-3

[81] Guerrero‐Vargas NN , Navarro‐Espindola R , Guzman‐Ruiz MA , et al. Circadian disruption promotes tumor growth by anabolic host metabolism; experimental evidence in a rat model. BMC Cancer. 2017;17(1):625. 28874144 10.1186/s12885-017-3636-3 PMC5585981

[82] Slat EA , Sponagel J , Marpegan L , et al. Cell‐intrinsic, bmal1‐dependent circadian regulation of temozolomide sensitivity in glioblastoma. J Biol Rhythms. 2017;32(2):121‐129. 28470120 10.1177/0748730417696788 PMC6410359

[83] Zhanfeng N , Yanhui L , Zhou F , Shaocai H , Guangxing L , Hechun X . Circadian genes Per1 and Per2 increase radiosensitivity of glioma in vivo. Oncotarget. 2015;6(12):9951‐9958. 25760074 10.18632/oncotarget.3179 PMC4496409

[84] Zhu L , Wang Q , Hu Y , Wang F . The circadian gene per1 plays an important role in radiation‐induced apoptosis and DNA damage in glioma. Asian Pac J Cancer Prev. 2019;20(7):2195‐2201. 31350984 10.31557/APJCP.2019.20.7.2195 PMC6745214

[85] Fan W , Caiyan L , Ling Z , Jiayun Z . Aberrant rhythmic expression of cryptochrome2 regulates the radiosensitivity of rat gliomas. Oncotarget. 2017;8(44):77809‐77818. 29100427 10.18632/oncotarget.20835 PMC5652816

[86] Wang F , Li C , Yongluo CL . The circadian gene clock plays an important role in cell apoptosis and the DNA damage response in vitro. Technol Cancer Res Treat. 2016;15(3):480‐486. 25976934 10.1177/1533034615585433

[87] Yu L , Wang Q , Wang C , et al. Design, synthesis, and biological evaluation of novel thienopyrimidine derivatives as PI3Kalpha inhibitors. Molecules. 2019;24(19):3422. 10.3390/molecules24193422 PMC6804295 31547116

[88] Jarabo P , de Pablo C , Gonzalez‐Blanco A , Casas‐Tinto S . Circadian gene cry controls tumorigenesis through modulation of myc accumulation in glioblastoma cells. Int J Mol Sci. 2022;23(4):2043. 35216153 10.3390/ijms23042043 PMC8874709

[89] Yu M , Li W , Wang Q , Wang Y , Lu F . Circadian regulator NR1D2 regulates glioblastoma cell proliferation and motility. Oncogene. 2018;37(35):4838‐4853. 29773903 10.1038/s41388-018-0319-8

[90] Gwon DH , Lee WY , Shin N , et al. BMAL1 suppresses proliferation, migration, and invasion of U87MG cells by downregulating cyclin B1, phospho‐AKT, and metalloproteinase‐9. Int J Mol Sci. 2020;21(7):235. 10.3390/ijms21072352 PMC7178273 32231148

[91] Jung CH , Kim EM , Park JK , et al. Bmal1 suppresses cancer cell invasion by blocking the phosphoinositide 3‐kinase‐Akt‐MMP‐2 signaling pathway. Oncol Rep. 2013;29(6):2109‐2113. 23563360 10.3892/or.2013.2381 PMC3694561

[92] Zhang F , Ren CC , Liu L , Chen YN , Yang L , Zhang XA . HOXC6 gene silencing inhibits epithelial‐mesenchymal transition and cell viability through the TGF‐beta/smad signaling pathway in cervical carcinoma cells. Cancer Cell Int. 2018;18:204. 30559605 10.1186/s12935-018-0680-2 PMC6290547

[93] Gao Y , Wu Y , Zhang N , et al. IDH1 gene mutation activates Smad signaling molecules to regulate the expression levels of cell cycle and biological rhythm genes in human glioma U87MG cells. Mol Med Rep. 2021;23(5):354. 33760141 10.3892/mmr.2021.11993 PMC7974315

[94] Guo Y , Hong W , Wang X , et al. MicroRNAs in microglia: how do MicroRNAs affect activation, inflammation, polarization of microglia and mediate the interaction between microglia and glioma? Front Mol Neurosci. 2019;12:125. 31133802 10.3389/fnmol.2019.00125 PMC6522842

[95] Zhou Q , Fu Q , Shaya M , Kugeluke Y , Li S , Dilimulati Y . Knockdown of circ_0055412 promotes cisplatin sensitivity of glioma cells through modulation of CAPG and Wnt/beta‐catenin signaling pathway. CNS Neurosci Ther. 2022;28(6):884‐896. 35332692 10.1111/cns.13820 PMC9062567

[96] Lecarpentier Y , Claes V , Vallee A , Hebert JL . Thermodynamics in cancers: opposing interactions between PPAR gamma and the canonical WNT/beta‐catenin pathway. Clin Transl Med. 2017;6(1):14. 28405929 10.1186/s40169-017-0144-7 PMC5389954

[97] Ma D , Hou L , Xia H , et al. PER2 inhibits proliferation and stemness of glioma stem cells via the Wnt/betacatenin signaling pathway. Oncol Rep. 2020;44(2):533‐542. 32468039 10.3892/or.2020.7624 PMC7336516

[98] Vallee A , Lecarpentier Y , Guillevin R , Vallee JN . Thermodynamics in gliomas: interactions between the canonical WNT/Beta‐catenin pathway and PPAR gamma. Front Physiol. 2017;8:352. 28620312 10.3389/fphys.2017.00352 PMC5451860

[99] Mohammad RM , Wu J , Azmi AS , et al. An MDM2 antagonist (MI‐319) restores p53 functions and increases the life span of orally treated follicular lymphoma bearing animals. Mol Cancer. 2009;8:115. 19958544 10.1186/1476-4598-8-115 PMC2794250

[100] Hayashi Y , Goyama S , Liu X , et al. Antitumor immunity augments the therapeutic effects of p53 activation on acute myeloid leukemia. Nat Commun. 2019;10(1):4869. 31653912 10.1038/s41467-019-12555-1 PMC6814808

[101] Ghilu S , Kurmasheva RT , Houghton PJ . Developing new agents for treatment of childhood cancer: challenges and opportunities for preclinical testing. J Clin Med. 2021;10(7):1504. 33916592 10.3390/jcm10071504 PMC8038510

[102] Sonkin D . Expression signature based on TP53 target genes doesn't predict response to TP53‐MDM2 inhibitor in wild type TP53 tumors. Elife. 2015;4:e10279. 26491944 10.7554/eLife.10279 PMC4728122

[103] Zhanfeng N , Chengquan W , Hechun X , et al. Period2 downregulation inhibits glioma cell apoptosis by activating the MDM2‐TP53 pathway. Oncotarget. 2016;7(19):27350‐27362. 27036047 10.18632/oncotarget.8439 PMC5053655

[104] Tseng TH , Shen CH , Huang WS , et al. Activation of neutral‐sphingomyelinase, MAPKs, and p75 NTR‐mediating caffeic acid phenethyl ester‐induced apoptosis in C6 glioma cells. J Biomed Sci. 2014;21:61. 24997497 10.1186/1423-0127-21-61 PMC4227122

[105] Goldsmith CS , Kim SM , Karunarathna N , et al. Inhibition of p38 MAPK activity leads to cell type‐specific effects on the molecular circadian clock and time‐dependent reduction of glioma cell invasiveness. BMC Cancer. 2018;18(1):43. 29316898 10.1186/s12885-017-3896-y PMC5761097

[106] Gowda P , Lathoria K , Sharma S , Patrick S , Umdor SB , Sen E . Rewiring of lactate‐interleukin‐1beta autoregulatory loop with clock‐bmal1: a feed‐forward circuit in glioma. Mol Cell Biol. 2021;41(9):e0044920. 34124933 10.1128/MCB.00449-20 PMC8384065

[107] Wang J , Li S , Li X , et al. Circadian protein BMAL1 promotes breast cancer cell invasion and metastasis by up‐regulating matrix metalloproteinase9 expression. Cancer Cell Int. 2019;19:182. 31346317 10.1186/s12935-019-0902-2 PMC6636133

[108] Sauzeau V , Beignet J , Vergoten G , Bailly C . Overexpressed or hyperactivated Rac1 as a target to treat hepatocellular carcinoma. Pharmacol Res. 2022;179:106220. 35405309 10.1016/j.phrs.2022.106220

[109] Gonzalez N , Cardama GA , Chinestrad P , et al. Computational and in vitro pharmacodynamics characterization of 1A‐116 Rac1 Inhibitor: relevance of Trp56 in its biological activity. Front Cell Dev Biol. 2020;8:240. 32351958 10.3389/fcell.2020.00240 PMC7174510

[110] Langston RG , Rudenko IN , Kumaran R , et al. Differences in stability, activity and mutation effects between human and mouse leucine‐rich repeat kinase 2. Neurochem Res. 2019;44(6):1446‐1459. 30291536 10.1007/s11064-018-2650-4 PMC6450775

[111] Cardama GA , Gonzalez N , Ciarlantini M , et al. Proapoptotic and antiinvasive activity of Rac1 small molecule inhibitors on malignant glioma cells. Onco Targets Ther. 2014;7:2021‐2033. 25378937 10.2147/OTT.S67998 PMC4218912

[112] Trebucq LL , Cardama GA , Lorenzano Menna P , Golombek DA , Chiesa JJ , Marpegan L . Timing of novel drug 1A‐116 to circadian rhythms improves therapeutic effects against glioblastoma. Pharmaceutics. 2021;13(7):1091. 34371781 10.3390/pharmaceutics13071091 PMC8309043

[113] Benvenga S , Feldt‐Rasmussen U , Bonofiglio D , Asamoah E . Nutraceutical supplements in the thyroid setting: health benefits beyond basic nutrition. Nutrients. 2019;11(9):2214. 10.3390/nu11092214 PMC6770945 31540254

[114] Li Y , Lv Y , Bian C , You X , Deng L , Shi Q . A Comparative genomic survey provides novel insights into molecular evolution of l‐aromatic amino acid decarboxylase in vertebrates. Molecules. 2018;23(4):917. 10.3390/molecules23040917 PMC6017361 29659490

[115] Zhang Y , Fan Y , Rui C , et al. Melatonin improves cotton salt tolerance by regulating ROS scavenging system and Ca(2 +) signal transduction. Front Plant Sci. 2021;12:693690. 34262587 10.3389/fpls.2021.693690 PMC8273866

[116] Akbarzadeh M , Movassaghpour AA , Ghanbari H , et al. The potential therapeutic effect of melatonin on human ovarian cancer by inhibition of invasion and migration of cancer stem cells. Sci Rep. 2017;7(1):17062. 29213108 10.1038/s41598-017-16940-y PMC5719004

[117] Lai SW , Liu YS , Lu DY , Tsai CF . Melatonin modulates the microenvironment of glioblastoma multiforme by targeting sirtuin 1. Nutrients. 2019;11(6):1343. 10.3390/nu11061343 PMC6627125 31207928

[118] Fatouh AM , Elshafeey AH , Abdelbary A . Agomelatine‐based in situ gels for brain targeting via the nasal route: statistical optimization, in vitro, and in vivo evaluation. Drug Deliv. 2017;24(1):1077‐1085. 28745530 10.1080/10717544.2017.1357148 PMC8241098

[119] Baltatu OC , Senar S , Campos LA , Cipolla‐Neto J . Cardioprotective melatonin: translating from proof‐of‐concept studies to therapeutic use. Int J Mol Sci. 2019;20(18):4342. 10.3390/ijms20184342 PMC6770816 31491852

[120] Franz T , Skopp G , Musshoff F . Findings of illicit drugs in hair of children at different ages. Int J Leg Med. 2021;135(2):465‐471. 10.1007/s00414-020-02479-7 33392654

[121] Xiong Y , Li M , Bai J , Sheng Y , Zhang Y . High level of METTL7B indicates poor prognosis of patients and is related to immunity in glioma. Front Oncol. 2021;11:650534. 33996568 10.3389/fonc.2021.650534 PMC8117938

[122] Sarma A , Sharma VP , Sarkar AB , Sekar MC , Samuel K , Geusz ME . The circadian clock modulates anti‐cancer properties of curcumin. BMC Cancer. 2016;16(1):759. 27680947 10.1186/s12885-016-2789-9 PMC5041585

[123] Damato AR , Katumba RGN , Luo J , et al. A randomized feasibility study evaluating temozolomide circadian medicine in patients with glioma. Neurooncol Pract. 2022;9(3):193‐200. 35601970 10.1093/nop/npac003 PMC9113320

[124] Wang X , Wang Y , Xie F , et al. Norepinephrine promotes glioma cell migration through up‐regulating the expression of Twist1. BMC Cancer. 2022;22(1):213. 35219305 10.1186/s12885-022-09330-9 PMC8882280

[125] Wagner PM , Monjes NM , Guido ME . Chemotherapeutic effect of SR9009, a REV‐ERB agonist, on the human glioblastoma T98G cells. ASN Neuro. 2019;11:1759091419892713. 31825658 10.1177/1759091419892713 PMC6909277

[126] Morioka N , Sugimoto T , Tokuhara M , Dohi T , Nakata Y . Noradrenaline induces clock gene Per1 mRNA expression in C6 glioma cells through beta(2)‐adrenergic receptor coupled with protein kinase A ‐ cAMP response element binding protein (PKA‐CREB) and Src‐tyrosine kinase ‐ glycogen synthase kinase‐3beta (Src‐GSK‐3beta). J Pharmacol Sci. 2010;113(3):234‐245. 20595783 10.1254/jphs.10031fp

[127] Seo YJ , Kwon MS , Shim EJ , Lee JY , Suh HW . The effects of phorbol 12‐myristate 13‐acetate, cholera toxin, prostaglandin E2 and norepinephrine on inducible nitric oxide synthase activation induced by lipopolysaccharide in C6 cells. Pharmacology. 2006;78(4):178‐184. 17047412 10.1159/000096349

[128] Sulli G , Rommel A , Wang X , et al. Pharmacological activation of REV‐ERBs is lethal in cancer and oncogene‐induced senescence. Nature. 2018;553(7688):351‐355. 29320480 10.1038/nature25170 PMC5924733

[129] Sapienza LG , Nasra K , Berry R , Danesh L , Little T , Abu‐Isa E . Clinical effects of morning and afternoon radiotherapy on high‐grade gliomas. Chronobiol Int. 2021;38(5):732‐741. 33557650 10.1080/07420528.2021.1880426

[130] Haraguchi S , Kamata M , Tokita T , et al. Light‐at‐night exposure affects brain development through pineal allopregnanolone‐dependent mechanisms. Elife. 2019;8:e45306. 31566568 10.7554/eLife.45306 PMC6850767

[131] Wianny F , Dzahini K , Fifel K , et al. Induced cognitive impairments reversed by grafts of neural precursors: a longitudinal study in a macaque model of parkinson's disease. Adv Sci (Weinh). 2022;9(10):e2103827. 35137562 10.1002/advs.202103827 PMC8981458

[132] Koopman T , Verburg N , Pouwels PJ , et al. Quantitative parametric maps of O‐(2‐[(18)F]fluoroethyl)‐L‐tyrosine kinetics in diffuse glioma. J Cereb Blood Flow Metab. 2020;40(4):895‐903. 31122112 10.1177/0271678X19851878 PMC7074601

[133] Stadlbauer A , Oberndorfer S , Zimmermann M , et al. Physiologic MR imaging of the tumor microenvironment revealed switching of metabolic phenotype upon recurrence of glioblastoma in humans. J Cereb Blood Flow Metab. 2020;40(3):528‐538. 30732550 10.1177/0271678X19827885 PMC7026844

